# CDK5RAP3 inhibits angiogenesis in gastric neuroendocrine carcinoma by modulating AKT/HIF-1α/VEGFA signaling

**DOI:** 10.1186/s12935-019-0997-5

**Published:** 2019-11-07

**Authors:** Jian-Xian Lin, Xiong-Feng Weng, Xin-Sheng Xie, Ning-Zi Lian, Sheng-Liang Qiu, Jia-Bin Wang, Jun Lu, Qi-Yue Chen, Long-Long Cao, Mi Lin, Ru-Hong Tu, Ying-Hong Yang, Si-Jia Liu, Min Hu, Yi-Ke Lin, Chang-Ming Huang, Chao-Hui Zheng, Ping Li, Jian-Wei Xie

**Affiliations:** 1 Department of Gastric Surgery, Fujian Medical University Union Hospital, No. 29 Xinquan Road, Fuzhou, 350001 Fujian China; 2 Key Laboratory of Ministry of Education of Gastrointestinal Cancer, Fujian Medical University, Fuzhou, 350108 Fujian China; 3 Fujian Key Laboratory of Tumor Microbiology, Fujian Medical University, Fuzhou, 350108 Fujian China; 4 Department of Pathology, Fujian Medical University Union Hospital, Fuzhou, 350001 Fujian China

**Keywords:** CDK5RAP3, VEGFA, Angiogenesis, Gastric neuroendocrine carcinoma

## Abstract

**Background:**

Angiogenesis plays critical roles in the progression and metastasis of malignant tumors. Gastric neuroendocrine carcinoma is an uncommon stomach cancer that is rich in blood vessels and exhibits highly malignant biological behavior with a poor prognosis. The role of CDK5RAP3 in GNEC has not been reported to date.

**Methods:**

Immunohistochemistry was used to assess the expression of CDK5RAP3 in GNEC tissues and adjacent non-tumor tissues. Cell lines with stable overexpression or knockdown of CDK5RAP3 were constructed using lentiviral transfection. Wound-healing assays, invasion and metastasis assays, tube formation assays, and tumor xenograft transplantation assays were performed to evaluate the effect of CDK5RAP3 on GNEC angiogenesis in vitro and in vivo. Real-time PCR, ELISA, western blot analysis, and confocal-immunofluorescence staining were used to explore the molecular mechanism of CDK5RAP3′s effect on angiogenesis.

**Results:**

Compared with their respective adjacent non-tumor tissues, protein levels of CDK5RAP3 were significantly decreased in GNEC tissues. Furthermore, low expression of CDK5RAP3 was correlated with more advanced TNM stage, increased tumor microvessel density, and poor prognosis. Functionally, we found that GNEC cells with CDK5RAP3 knockdown promoted human umbilical vein endothelial cells migration and tube formation via activation of AKT/HIF-1α/VEGFA signaling, resulting in increased levels of VEGFA in GNEC cell supernatant. In addition, CDK5RAP3 overexpression in GNEC cells caused the opposing effect. Consistent with these results, nude mouse tumorigenicity assays showed that CDK5RAP3 expression downregulated angiogenesis in vivo. Lastly, patients with low CDK5RAP3 expression and high VEGFA expression exhibited the worst prognosis.

**Conclusions:**

This study demonstrated that CDK5RAP3 inhibits angiogenesis by downregulating AKT/HIF-1α/VEGFA signaling in GNEC and improves patient prognosis, suggesting that CDK5RAP3 could be a potential therapeutic target for GNEC.

## Background

Neuroendocrine neoplasms (NENs) exclusively made by cells with a neuroendocrine phenotype, i.e., expressing markers of neuroendocrine differentiation like synaptophysin (SYN), and chromogranin A (CgA), neuron specific enolase (NSE) and others including hormones. As such, neuroendocrine neoplasms (NENs) may develop at any anatomical site [1]. Gastric NENs represent about 7–8% of all NETs [2]. In 2010, the WHO named G3 neuroendocrine neoplasms with a cell mitotic rate > 20 or Ki-67 index > 20% “neuroendocrine carcinomas” (NECs) [3]. Gastric neuroendocrine carcinoma (GNEC) reportedly accounts for approximately 15–20% of gastric NENs [4]. GNEC is usually poorly differentiated with a rich blood vessel supply that exhibits highly malignant biological behaviors. Lymph node metastasis occurs in greater than 70% of cases, and approximately 50% of patients experience liver metastases [5]. With improvements in diagnostic technology, the incidence of GNEC has gradually increased [6], but clinical treatment of these diseases remains poor [7]. Therefore, examining the molecular mechanism of GNEC is of great significance for identifying new therapeutic strategies.

Increasing numbers of studies have verified that dysregulation of angiogenesis is associated with cancer progression and metastasis, and anti-angiogenic treatments have been an important anti-cancer strategy [8]. Angiogenesis, including vascular endothelial cell migration, aggregation, and new tube formation, is regulated by multiple growth factors [9]. Among these growth factors, vascular endothelial growth factor A (VEGFA) plays the most important role [10]. Normally, VEGFA secretion is strictly controlled, and when VEGFA levels are dysregulated, abnormal angiogenesis occurs [8]. Hoshino et al. [11] found that many cancer cells promote the secretion of angiogenic factors, including VEGFA, to induce tumor angiogenesis. However, the upstream mechanism by which GNEC cells regulate VEGFA expression has not been thoroughly elucidated to date.

Cyclin dependent kinase 5 regulatory subunit associated protein 3 (CDK5RAP3, or C53 for short) and was first identified as a binding protein for CDK5 activator proteins p35 and p39 [12]. CDK5RAP3 expression varies significantly in different human tumors. CDK5RAP3 is upregulated in hepatocellular carcinoma, colon adenocarcinoma and lung cancer [13, 14] while being downregulated in human head and neck squamous cell carcinomas (HNSCCs) [15]. Our previous study demonstrated that CDK5RAP3 expression was downregulated in gastric cancer, resulting in inhibition of Wnt/β-catenin signaling [16, 17]. However, the role of CDK5RAP3 in GNEC has not been thoroughly elucidated to date.

In this study, we found that CDK5RAP3 expression was significantly decreased in GNEC tissues, which correlated with increased angiogenesis. GNEC cells with reduced expression of CDK5RAP3 promoted HUVEC migration and tube formation via activation of AKT/HIF-1α/VEGFA signaling, increasing the level of VEGFA in GNEC cell supernatant. Therefore, blocking this signaling cascade may represent a potential therapeutic approach for the treatment of GNEC.

## Methods

### Human gastric neuroendocrine carcinoma tissues

The human gastric neuroendocrine carcinoma tissues of 59 patients were obtained from Fujian Medical University Union Hospital (Fujian, China) with detailed clinic pathologic parameters. All gastric neuroendocrine carcinomas were diagnosed according to histopathological examination by two experienced pathologists based on histological specimens after gastrectomy. All patients underwent gastrectomy with D2 lymph node dissection from 2011 to 2017. None of the patients underwent preoperative chemotherapy or radiotherapy. Postoperative adjuvant chemotherapy was performed with 5-fluorouracil-based drugs plus oxaliplatin in advanced cases. The pathologic stage of the tumor was re-assessed according to the 2010 Union for International Cancer Control (UICC) TNM classification (seventh edition) [18]. The respective adjacent non-tumor tissues were located at least 5 cm from the gastric neuroendocrine carcinoma. All fresh specimens were stored in liquid nitrogen after resection until protein extraction. The 51 paraffin-embedded GNEC tissues were collected for Immunohistochemistry (IHC) from February 2011 to December 2017, and all of them had respective adjacent non-tumor tissues. The 8 fresh gastric neuroendocrine carcinoma tissues and respective adjacent non-tumor tissues were recruited in 2017, This study was approved by the ethics committee of Fujian Medical University Union Hospital and written consent was obtained from all patients involved.

### Follow-up

After surgery, all patients were followed by outpatient visits, telephone calls and letters. Follow-up was conducted every 3 months in the first 2 years, every 6 months in the next 3 years, and every year afterwards or until death. The survival time was the time from the date of surgery until the last contact, or the date of death. The deadline for follow-up was December 2018. All 51 patients involved in the IHC analysis were followed up with and none were lost.

### Immunohistochemistry

Paraffin blocks that contained sufficient formalin-fixed tumor specimens were serial sectioned at 4 μm and mounted on silane-coated slides for IHC analysis. The sections were deparaffinized with dimethylbenzene and rehydrated through 100, 100, 95, 85 and 75% ethanol. Antigen retrieval treatment was done in an autoclave at 121 °C for 2 min in 0.01 mol/l sodium citrate buffer (pH 6.0) and endogenous peroxidase was blocked by 3% H^2^O^2^ incubation in for 10 min at room temperature. The slides were then washed in PBS and blocked with 10% goat serum (ZhongShan Biotechnology, China) for 30 min, incubated with anti-human CDK5RAP3 (ab157203, 1:100 dilution; Abcam) or VEGFA (ab1316, 1:200 dilution; Abcam), CD31 (77699, 1:100 dilution; 3528, 1:1600 dilution; CST) antibody in a humidified chamber at 4 °C overnight. Following three additional washes in PBS, the sections were incubated with HRP-conjugated secondary antibody for 30 min at room temperature. Next, the visualization signal was developed with diaminobenzidine (DAB) solution and all of the slides were counterstained with 20% hematoxylin. Last the slides were dehydrated and mounted with cover slips. For negative controls, the primary antibody diluent was used in place of primary antibody.

### Evaluation of immunostaining intensity

The IHC-stained tissue sections were reviewed under microscope by 2 pathologists who were blinded to the clinical parameters, and scored independently according to the intensity of cellular staining and the proportion of stained tumor cells. The CDK5RAP3 and VEGFA proteins were immunohistochemically stained yellowish to brown in the cytoplasm, and displayed all or none mode in tumor tissues. The degree of immunostaining of indicated proteins was evaluated and scored by 2 independent observers. Briefly, each sample was scored according to staining intensity (no staining = 0; weak staining = 1; moderate staining = 2; strong staining = 3) and the number of stained cells (0% = 0; 1–25% = 1; 26–50% = 2; ≥ 51% = 3). The staining index (SI) was calculated as the product of staining intensity × percentage of positive tumor cells, result in scores of 0, 1, 2, 3, 4, 6, and 9. The cutoff for high expression was set as SI scores greater than 3, whereas SI scores less than or equal to 3 were considered to be low expression [16, 19]. The microvessel density (MVD) was evaluated by CD31 immunohistochemistry staining. MVD was calculated as the average count of microvessel in the 4 hot spots at high magnification (200×) [20].

### Cell culture

Human GNEC cell lines ECC10 and ECC12 were purchased from RIKEN BRC CELL BANK (3-1-1 Koyadai, Tsukuba, Ibaraki, 305-0074 Japan) which were both derived from small-cell gastric carcinoma [21] and reported by several literatures [22–24]. Human umbilical vein endothelial cells (HUVECs) were obtained from the Cell Line Bank, Chinese Academy of Sciences. All cells were tested to be free of mycoplasma by MycoAlert Mycoplasma Detection kit (Lonza). We don’t have normal neuroendocrine cells in cell banks. We could not compare the expression levels of CDK5RAP3 in these two cell lines with normal. According to the revised World Health Organization (WHO) criteria (2015) for the pathological diagnosis of neuroendocrine carcinoma, neuroendocrine markers must be examined by immunohistochemistry (IHC). Immunohistochemical neuroendocrine markers, such as CD56, synaptophysin (SYN), and chromogranin A (CgA), can be helpful in diagnosing neuroendocrine tumors. ECC10 and ECC12 were identified by Southern blotting and cell histochemistry of SYN and CgA. ECC12 was positive for SYN and CgA, ECC10 was positive for SYN and negative for CgA, and Human gastric cancer cell lines AGS and MGC-803 were negative for SYN and CgA (Additional file 1: Figure S1A–C). ECC10 and ECC12 were cultured in RPMI 1640 (Gibco, Grand Island, NY) while HUVECs were cultured in DMEM (Gibco, Grand Island, NY) supplemented with 10% fetal bovine serum (FBS) (Gibco, Grand Island, NY). All cells were maintained at 37 °C in a humidified incubator with 5% CO^2^ condition.

### Establishment of cell lines

Lentiviral constructs of CDK5RAP3 (NM_176096) shRNA (Lenti-shC53), CDK5RAP3 overexpression (Lenti-C53), and their corresponding empty vectors Lenti-scramble and Lenti-empty (Lenti-scr and Lenti-emp), CDK5RAP3 splice variants synonymous mutant (Lenti-C53 mut) [16], and their matched empty vectors (Lentictrl mut) were purchased from Shanghai Genechem Co. Ltd. ECC10 and ECC12 cells were seeded in 6-well plates at a concentration of 3 × 10^5^ cells per well (30–40% confluence) on the day before lentivirus transduction. Lenti-shC53 and Lenti-C53 were transduced into cells at a suitable multiplicity of infection using polybrene (10 mg/ml) and Enhanced Infection Solution (Genechem, China). The matched empty vectors (Lenti-scr and Lenti-emp) were simultaneously transduced into cells using the same methods to control for the impact of the viral vector. After incubation for 6–8 h, the medium was replaced with fresh medium. To establish stable cell lines, the cells were selected with the corresponding antibiotic puromycin at 2–3 mg/ml (Sigma) for at least 1 week, 48 h after transfection. At the indicated time points, the cells were harvested for mRNA and protein analysis as well as other assays. The sequences of shC53 were designed and chemically synthesized as: Forward 5′-TGGGAAACTCAACGGTGTACTTCAAGAGAGTACACCGTTGAGTTTCCCTTTTTTC-3′; Reverse 3′-TCGAGAAAAAAGGGAAACTCAACGGTGTACTCTCTTGAAGTACACCGTTGAGTTTCCCA-5′.

### Preparation of tumor conditioned medium (TCM)

The same amount of ECC10 or ECC12 cells which were transfected with Lenti-shC53, Lenti-C53, Lenti-scr or Lenti-emp were seeded into a 6-cm dish with 10% fetal bovine serum and cultured for 6–8 h. Then, the cells were washed with 0.01 M phosphate-buffered saline (PBS) three times and cultured with 5 ml serum-free RPMI-1640 for 24 h. TCM was collected after centrifugation at 12,000*g* for 10 min at 4 °C and then stored at − 80 °C until used.

### Enzyme-linked immunoassay (ELISA)

VEGFA in the medium was measured by using the Human VEGFA ELISA kit (ab119566, abcam, USA) according to manufacturer’s instruction. Standard curves were created using purified VEGFA and the CurveExpert 1.4 software program.

### Wound-healing assay

HUVECs were seeded into 6-well plates. When the cells reached confluence, scrape wounds were made in each well. Then, the cells were washed with 0.01 M phosphate-buffered saline (PBS) three times and cultured with TCM. The cells were photographed at the indicated time points.

### HUVECs recruitment assay

To evaluate GNEC cell-mediated HUVECs recruitment, a 24-well transwell assay was used in our study, which were performed in Transwell chambers with Matrigel coated pore membrane (polycarbonate flters of 8-mm porosity; BD Biosciences, Franklin Lakes, NJ, USA). HUVECs with 200 μl serum-free RPMI-1640 were seeded in the upper chamber (5 × 10^4^ cells/well), The lower chamber was loaded with 600 μl TCM containing 20% FBS. The chambers were incubated for 12 h at 37 °C, cells that did not migrate or invade were removed with a cotton swab. The migrated cells were fixed in methanol for 10 min and stained with crystal violet for 5 min. Cells in 10 random microscopic fields (×100 magnification) for each insert were counted.

### HUVEC tube formation assay

HUVECs were suspended in TCM and seeded on a 96-well plate coated with matrigel (100 μl/well, BD Biosciences). After 6 h of incubation at 37 °C, tube formation was observed and photographed with a computer-assisted inverted microscope (Nikon). Ten random fields per sample were photographed at ×100 magnification. The number of branch points of the connected tubes was counted and compared between different groups.

### Immunofluorescence staining

Cells were grown on glass coverslips, washed twice with PBS, fixed with PBS containing 4% formaldehyde at 4 °C for 10 min, and permeabilized with 0.2% Triton X-100 in PBS at 4 °C for 10 min. Following washing with PBS, cells were blocked with 10% goat serum (Abcam, Cambridge, MA, USA) at room temperature for 2 h. Then cells were incubated overnight at 4 °C with primary antibody against VEGFA(ab1316,1:200 dilution; abcam)washed with PBS and incubated with secondary antibody Alexa Fluor^®^ 568 IgG (Invitrogen; Thermo Fisher Scientifc, USA) The nucleus was stained with DAPI (Sigma-Aldrich; Merck KGaA) at 37 °C for 1 min. The coverslips were mounted with the SlowFade^®^ Gold reagent (Invitrogen; Thermo Fisher Scientific, Inc.) and observed under a laser confocal scanning microscope.

### Western blot

Total protein was extracted from cells with RIPA lysis buffer (Biyotime, China) containing protease inhibitors. The protein concentration of the lysates was analyzed by BCA protein assays (Thermo Fisher Scientific, USA). 40 μg of protein was separated on a 10% SDS-polyacrylamide gel and blotted onto polyvinylidene difluoride (PVDF) membranes (Millipore, USA). After blocking with 5% bovine serum albumin (BSA) for 1 h, the membranes were then incubated with primary antibodies [CDK5RAP3 (ab157203, Abcam, USA); VEGFA (ab6154, abcam, USA); HIF-1α (ab1, Abcam, USA);p-AKT (S473) (4060, CST, USA), AKT (9272, CST, USA)] overnight at 4 °C and horseradish peroxidase-conjugated secondary antibodies for 1 h at room temperature. Immunoreactive signals were detected using the ECL detection system. Immunoblotting of glyceraldehyde-3-phosphate dehydrogenase (GAPDH) was performed as an internal control.

### RNA isolation and real-time RT-PCR

Total RNA was extracted using Trizol reagent (Takara, Dalian, China). A 4 μg template RNA was used to synthesize the first-strand cDNA using a Prime Script RT reagent kit (RR047A TakaraBio, Tokyo, Japan). Quantitative real-time RT-PCR analysis was performed using SYBR^®^ Fast qPCR Mix (TaKaRa, Shiga, Japan) and a Agilent Technologies Stratagene Mx3000p Real-Time System (Agilent, USA) with SYBR Green to determine the mRNA expression level of a gene of interest. A reference gene, glyceraldehyde-3-phosphate dehydrogenase (GAPDH), was used to normalize gene expression levels. The mRNA abundance was analyzed using the comparative threshold cycle (2^−ΔΔCT^) method. The primers used in the RT-PCR were as follows: CDK5RAP3: forward: 5′-ATTTTTGGCCGATACTCTTCACA-3′; reverse: 5′-TCATAGTTGACATTCCGAACCAG-3′; VEGFA: forward: 5′-CATGAACTTTCTGCTGTCTTGG-3′; reverse: 5′-CATTTGTTGTGCTGTAGGAAGC-3′; GAPDH: forward 5′-AAGGTGAAGGTCGGAGTCAA-3′; reverse 5′-CCATGTAGTTGAGGTCAATGAAGG-3′. All samples were measured with at least three independent experiments and the results are expressed as the mean ± SD of the comparative analysis.

### Tumor xenograft transplantation assay

SPF-grade male BALB/c nude mice were purchased from the Institute of Zoology, Chinese Academy of Sciences. All animal work procedures were performed in accordance with the Animal Care Committee of Fujian Medical University, China. For xenograft animal experiments, 7.0 × 10^6^ CDK5RAP3 stable knockdown, overexpressing ECC12 cells and respective control cells were resuspended in 100 μl phosphate-buffered saline (PBS) and mixed it with Matrigel in a ratio of 1:1, and subcutaneously injected into the right flank of the mice. Tumor volume was measured every 7 days and calculated by the formula: (length × width^2^)/2. Mice were sacrificed 8 weeks after injection and tumor tissues were surgically excised from the mice. Tumor weight was evaluated. Part of the tumor tissues were used for protein extraction, and the rest were fixed in 4% paraformaldehyde and embedded in paraffin. Five-micrometer-thick sections were prepared for immunohistochemical staining.

### Statistical analysis

All statistical analyses were performed using SPSS 22.0 for Windows (SPSS, Chicago, IL) and Prism 5.0 software (GraphPad). Select the appropriate test method based on the type of the variable and the purpose of the comparison. The chi-squared test was used to evaluate the difference in proportions, and student’s t-tests were used to evaluate continuous variables. The Kaplan–Meier method was used to calculate the survival rate and the survival curve was drawn. The survival rate was compared by Log-rank test. P < 0.05 was considered statistically significant and all P values were two-sided.

## Results

### Low expression of CDK5RAP3 in GNEC tissues, especially in high microvessel density areas, is correlated with poor prognosis

**Fig. 1:**
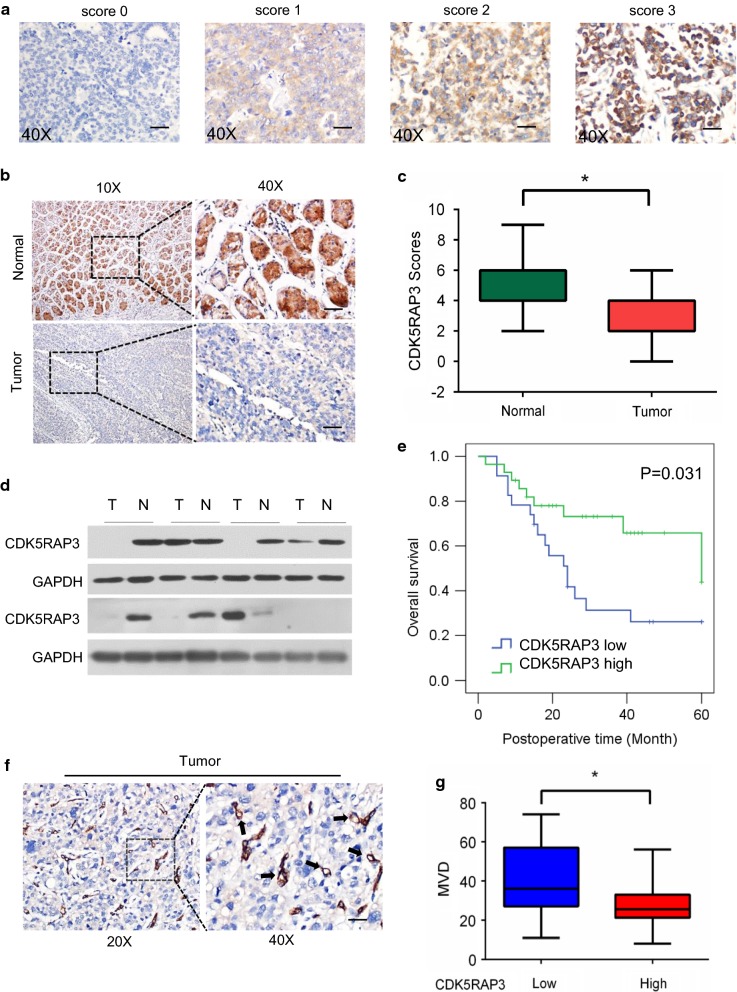
Expression and prognostic value of CDK5RAP3 in GNEC. **a** Immunohistochemical staining of CDK5RAP3 expression in GNEC tissues and criteria for immunohistochemistry scoring. Score 0: no staining, Score 1: weak staining, Score 2: moderate staining, Score 3: strong staining. Each section was examined under a high-power field (40×). **b** Representative image of CDK5RAP3 protein expression in GNEC tissues and adjacent normal gastric tissues analyzed using IHC. **c** IHC scores of CDK5RAP3 in GNEC tissues and adjacent normal gastric tissues was compared using Student’s t-test. n = 51 (*P < 0.05). **d** Protein levels of CDK5RAP3 in GNEC tissues and adjacent normal gastric tissues from 8 additional patients measured by western blot analysis. **e** Kaplan–Meier survival curve of gastric cancer patients with “CDK5RAP3 high” and “CDK5RAP3 low” expression levels (P < 0.05, log-rank test). CD31 staining was used to determine MVD. **f** Representative images microvessel staining (CD31+) under high-power fields (20×, 40×). **g** MVD was calculated as the average measurement of 10 random high-power fields (20×). Comparison of MVD in GNEC tissues stratified by different CDK5RAP3 levels. Low CDK5RAP3 tissues: 39 ± 3.75 per field, n = 23 cases; High CDK5RAP3 tissues: 28 ± 1.94 per field, n = 28 cases, P < 0.01. Scale bar, 50 µm

To examine the expression pattern of CDK5RAP3 in GNEC, CDK5RAP3 protein expression was analyzed in 51 GNEC tissues and adjacent normal gastric tissues using IHC. Scoring standards are shown in Fig. 1a. CDK5RAP3 expression score was significantly lower in tumor tissues compared to their respective non-tumor tissues (Fig. 1b, c). In addition, CDK5RAP3 protein levels were detected in GNEC tissues and respective adjacent non-tumor tissues from 8 additional patients using western blot analysis. CDK5RAP3 protein levels were downregulated in 5 of 8 (62.5%) GNEC tissues compared to paired normal tissues (Fig. 1d). These data indicate that CDK5RAP3 is downregulated in GNEC tissues. Further analysis of 51 GNEC patient clinicopathological parameters revealed that CDK5RAP3 expression is associated with invasion depth, lymph node metastasis, and TNM stage (Table 1), suggesting that CDK5RAP3 expression is related to GNEC progression. Overall survival for patients with low CDK5RAP3 expression (26.1%) was reduced compared to patients with high CDK5RAP3 expression (43.8%) (Fig. 1e). A Cox proportional hazards regression model was created to examine the effect of CDK5RAP3 expression and other pathological parameters on patient prognosis. We performed IHC with anti-CD31 (vascular endothelial cell marker) in 51 GNEC tissues and quantified microvessel density (MVD). MVD was significantly increased in tissues with reduced CDK5RAP3 expression compared to tissues with high CDK5RAP3 expression (Fig. 1f, g). Taken together, these results indicate that downregulation of CDK5RAP3 is correlated with poor prognosis and increased MVD in GNEC.

**Table 1:** Relationships between CDK5RAP3 protein expressions in GNEC tissues and various clinicopathological characteristic

Variables	Total	C3 expression			
		Low	High	X^2^	P
Gender				0.221	0.638
Male	43	20	23		
Female	8	3	5		
Age (years)				0.634	0.426
> 60	34	14	20		
≤ 60	17	9	8		
Tumor size (cm)				3.804	0.051
> 5	35	19	16		
≤ 5	16	4	12		
Tumor location				4.819	0.090
Lower 1/3	9	7	2		
Middle 1/3	9	3	6		
Upper 1/3	33	13	20		
Histological type				2.003	0.157
Large cell type	32	12	20		
Small cell type	19	11	18		
Depth of invasion				22.203	< 0.001
T1	2	2	0		
T2	7	0	7		
T3	29	9	20		
T4	13	12	1		
Lymph node metastasis				4.104	0.043
N0	11	1	9		
N1–3	40	21	19		
Distant metastasis				2.534	0.111
Negative	49	21	28		
Positive	2	2	0		
TNM stage				5.368	0.021
I + II	20	5	15		
III + IV	31	18	13		

P < 0.05, statistical significance

^a^t-test

### CDK5RAP3 in GNEC cells indirectly affects vein endothelial cell migration and tube formation in vitro

**Fig. 2:**
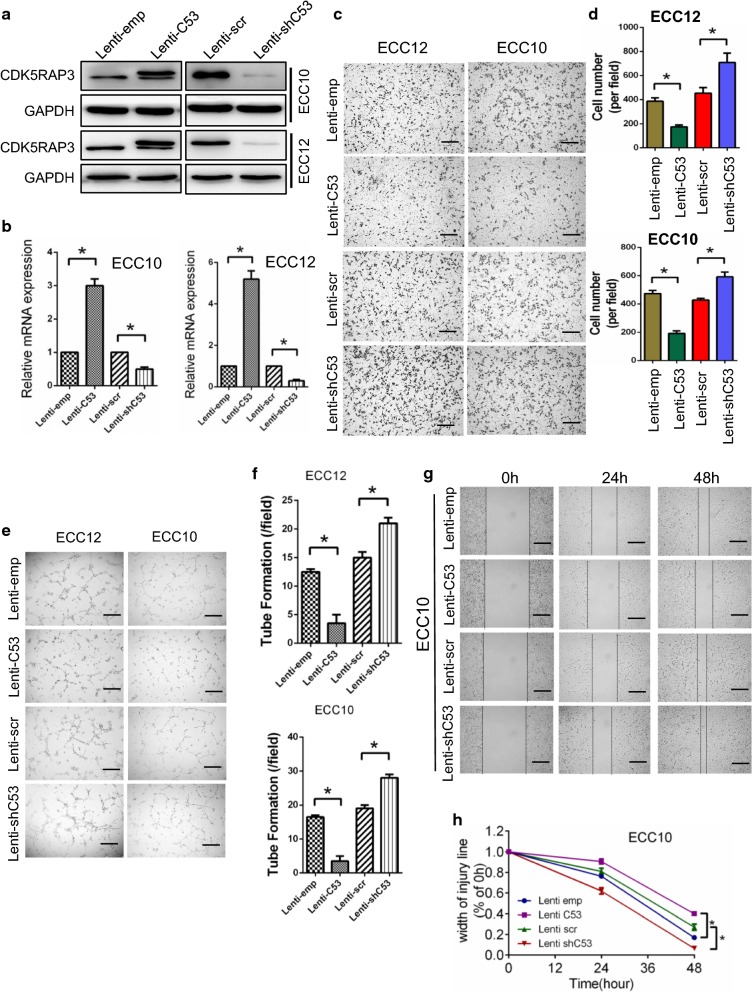
CDK5RAP3 in GNEC cells indirectly affects endothelial cell migration and tube formation in vitro. ECC10 and ECC12 cells with stable overexpression or knockdown of CDK5RAP3 were created. **a** CDK5RAP3 expression changes were confirmed using western blot analysis. CDK5RAP3 mRNA changes were confirmed using RT-PCR. **b** CDK5RAP3 inhibits tumor-induced HUVEC migration as shown by transwell migration assays. Representative images are shown under high-power fields (10×) (**c**). Quantification of results is presented in **d**. CDK5RAP3 inhibits tumor-induced HUVEC angiogenesis as shown by tube formation assays. Representative images are shown under high-power fields (10×) (**e**). Quantification of the results is presented in **f**. CDK5RAP3 in ECC10 cells indirectly inhibits tumor-induced HUVEC migration as shown by wound healing assay. Representative images are shown under high-power fields (10×) (**g**). Quantification of results is presented in **h**. Results show mean ± SD from at least three independent experiments. *P < 0.05, **P < 0.01, ***P < 0.001. Scale bar, 200 µm

We detected the basic expression of CDK5RAP3 from ECC10 and ECC12 by Western blot (Additional file 1: Figure S1D). The expression of CDK5RAP3 in ECC10 cells was lower than in ECC12 cells. To examine the effect of CDK5RAP3 on GNEC cells, ECC10 and ECC12 cells were created with stable overexpression or knockdown of CDK5RAP3. Changes in CDK5RAP3 expression were confirmed by both Western blot and RT-PCR (Fig. 2a, b). Because CDK5RAP3 was isolated as a binding protein of the Cdk5 activator p35, we performed Western blot to detect the expression of CDK5, p35 and p39 (Additional file 1: Figure S1E). The results showed that the expression levels of these proteins showed a trend consistent with CDK5RAP3. Since angiogenesis involves endothelial cell migration and tube formation, we performed HUVEC recruitment assays, as well as wound healing and tube formation assays, to gain insight into the role of CDK5RAP3 in GNEC. In brief, TCM from cells with stable overexpression or knockdown of CDK5RAP3 or control was collected and added to the lower chamber with the HUVECs in the upper chamber of the Transwell system. Compared to the control group, TCM from stably knocked down CDK5RAP3 cells exhibited significantly enhanced HUVEC cell migration (Fig. 2c, d) and tube formation potential (Fig. 2e, f). In contrast, TCM from stably overexpressed CDK5RAP3 cells exhibited decreased migration (Fig. 2c, d) and tube formation abilities (Fig. 2e, f). Similar patterns were observed in wound healing (Fig. 2g, h; Additional file 1: Figure S2A, B), suggesting that dysregulation of CDK5RAP3 in GNEC cells may play an indirect role in EC migration and tube formation in vitro.

### CDK5RAP3 in GNEC cells inhibits EC migration and tube formation by reducing secretion of VEGFA into the supernatant via suppression of AKT/HIF-1α/VEGFA signaling

**Fig. 3:**
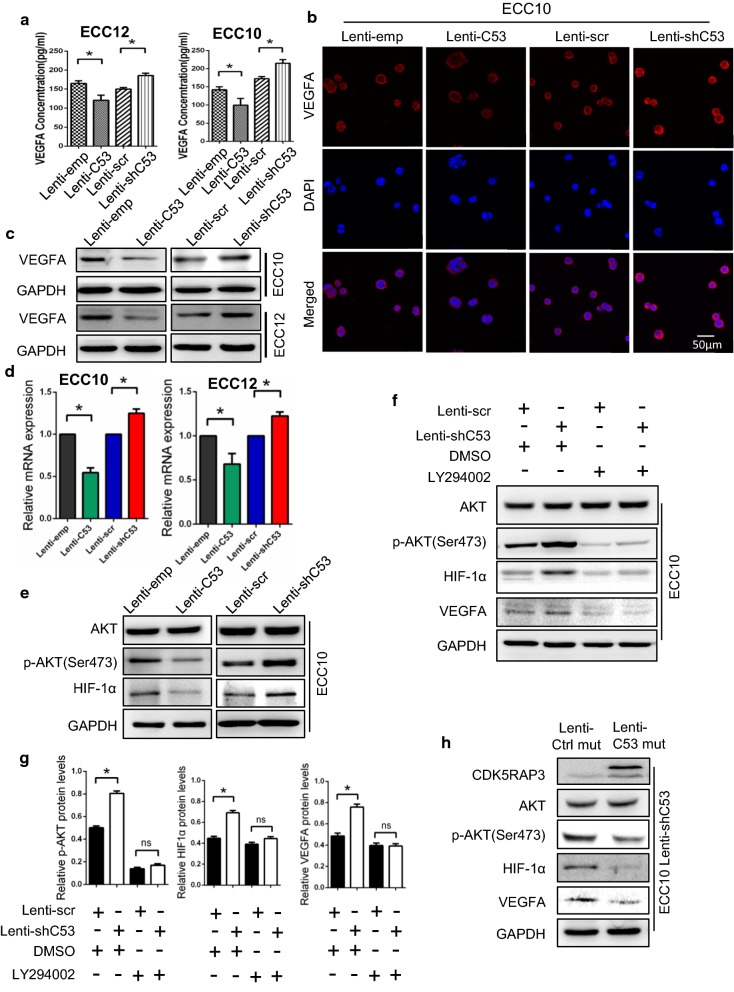
CDK5RAP3 in GNEC cells reduces secretion of VEGFA into the supernatant via suppression of AKT/HIF-1α/VEGFA signaling. **a** VEGFA levels in four stable ECC10/ECC12 cell supernatants as determined by ELISA. CDK5RAP3 in GNEC cells reduces secretion of VEGFA. **b** CDK5RAP3 decreases VEGFA protein levels in ECC10 cells as shown by immunofluorescence staining. Scale bar, 50 µm. **c** VEGFA expression in four stable ECC10/ECC12 cells detected by western blot analysis. CDK5RAP3 expression is inversely correlated with VEGFA protein expression. **d** mRNA levels of VEGFA in four stable ECC10/ECC12 cells measured by RT-PCR. **e** p-AKT (S473) and HIF-1α expression in ECC10 cells detected by western blot analysis. Knockdown of CDK5RAP3 increases protein levels of p-AKT (S473) and HIF-1α. Overexpression of CDK5RAP3 mediated opposing effects. **f** Lenti-scr and Lenti-shC53 transfected ECC10 cells were treated with 20 μM LY294002 (a highly selective inhibitor of AKT). Effects of AKT knockdown confirmed by western blot analysis. Inhibition of AKT decreases protein levels of HIF-1α and VEGFA in ECC10 cells. Quantification of results is presented in **g**. Mutant CDK5RAP3 reversed the trend in protein levels for p-AKT (S473), HIF-1α and VEGFA induced by CDK5RAP3 knockdown. Changes in CDK5RAP3 expression were confirmed by western blot analysis (**h**)

Next, we explored the mechanism whereby CDK5RAP3 inhibits HUVEC migration and tube formation in GNEC cells. We hypothesized that cytokines or growth factors may participate in this process. Since VEGFA is one of the most important growth factors in angiogenesis, VEGFA levels in Lenti-emp, Lenti-C53, Lenti-scr and Lenti-shC53 GNEC cell supernatants were determined by ELISA assay. As expected, higher levels of VEGFA were observed in CDK5RAP3 knockdown GNEC cell supernatant than in supernatant from control cells (Fig. 3a). In contrast, overexpression of CDK5RAP3 induced decreased VEGFA levels in the supernatant, suggesting that CDK5RAP3 inhibits VEGFA secretion in GNEC cells. We detected VEGFA protein levels from CDK5RAP3-overexpressing or knockdown GNEC cells using western blotting and immunofluorescence staining. Results revealed that overexpression of CDK5RAP3 reduced VEGFA protein levels, while knockdown of CDK5RAP3 significantly enhanced VEGFA protein levels (Fig. 3b, c; Additional file 1: Figure S3A). Similar results were obtained when VEGFA mRNA levels were examined using RT-PCR (Fig. 3d). These data confirm that VEGFA is regulated by CDK5RAP3 in GNEC cells at both post-transcriptional and post-translational levels.

Our recent work revealed that CDK5RAP3 inhibits phosphorylation of AKT at Ser473 in gastric cancer [17]. Activation of AKT promotes expression of VEGFA through multiple pathways [25–28]. In this study, western blot analysis of ECC10 cell lysates showed higher levels of both phospho-AKT (p-AKT) at Ser473 and HIF-1α in CDK5RAP3 knockdown cells than in control cells. In contrast, expression of CDK5RAP3 reduced levels of p-AKT (s473) and HIF-1α compared to control cells (Fig. 3e). Next, CDK5RAP3 stable knockdown ECC10 cells were treated with 20 μM Ly294002, a highly selective inhibitor of AKT, for 48 h. Levels of p-AKT were significantly reduced in response to Ly294002, and levels of HIF-1α, and VEGFA were reduced (Fig. 3f, g). To rule out off-target effects, we introduced a synonymous mutant in CDK5RAP3 that cannot be silenced by the Lenti-shC53. Overexpression of this CDK5RAP3 mutant reduced the levels of p-AKT (s473), HIF-1α, and VEGFA in stable knockdown CDK5RAP3 ECC10 cells (Fig. 3h). Taken together, these data indicate that reduced expression of CDK5RAP3 activates the AKT/HIF-1α/VEGFA axis in GNEC cells.

### CDK5RAP3 suppressed tumor growth and angiogenesis in vivo

**Fig. 4:**
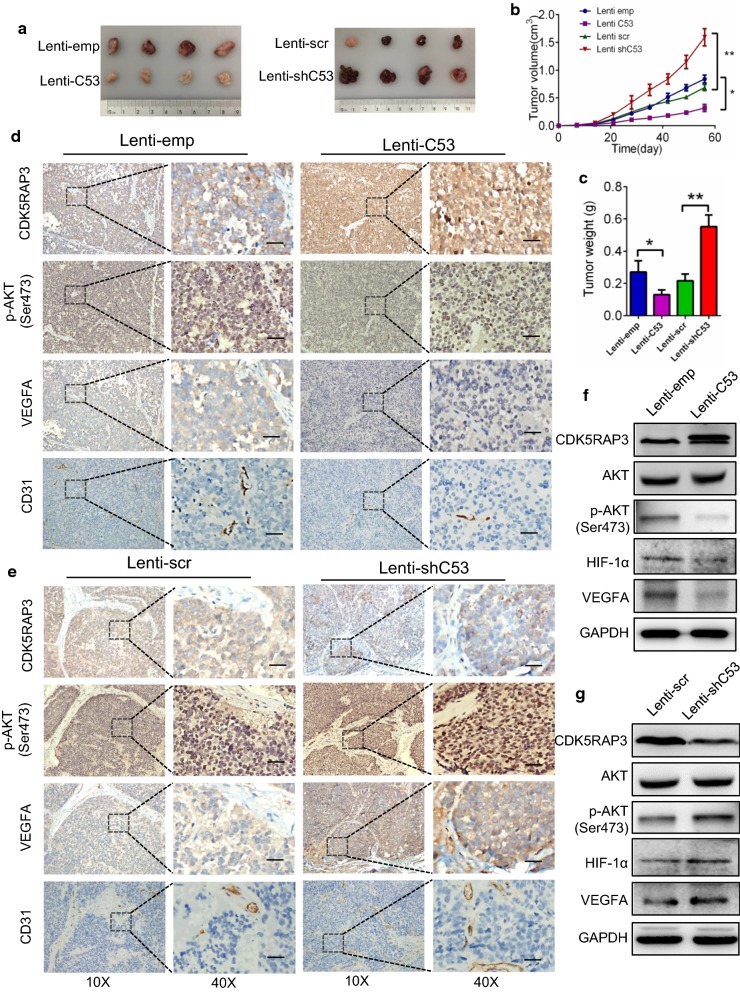
CDK5RAP3 suppresses tumor growth and angiogenesis in vivo. Xenograft models using the four stable ECC12 cells were created and allowed to grow for 8 weeks. **a** Representative images of the effect of CDK5RAP3 knockdown or CDK5RAP3 overexpression. **b** Tumor size was measured every 7 days until mice were sacrificed. **c** Average tumor weight of the four different groups. **d** Representative IHC staining of CDK5RAP3, p-AKT (S473), CD31 and VEGFA in xenograft tumor tissues. Mice injected with cells overexpressing CDK5RAP3 exhibited reduced MVD, p-AKT (S473), and VEGFA expression in tumor tissue compared to those injected with control cells. **e** Mice injected with CDK5RAP3 knockdown cells presented with increased MVD, p-AKT (S473), and VEGFA expression in tumor tissue compared to those injected with control cells. Protein levels of CDK5RAP3, HIF-1α, p-AKT (S473) and VEGFA were measured by western blotting in CDK5RAP3 overexpressing tumor tissues (**f**) and CDK5RAP3 knockdown tumor tissues (**g**). Scale bar, 50 µm

An ECC12 xenograft model was used to evaluate the effect of CDK5RAP3 on GNEC angiogenesis. Xenografts with high CDK5RAP3 expression grew more slowly than those with control cells. In contrast, xenografts with downregulated CDK5RAP3 expression grew much faster than control cells (Fig. 4a–c). IHC staining of CD31, p-AKT (s473), CDK5RAP3 and VEGFA in xenograft tumor tissues showed that MVD in the CDK5RAP3 overexpression group was lower than in the control group. The expression of VEGFA and p-AKT (s473) was decreased compared to the control group (Fig. 4d). In contrast, the vascular density in the CDK5RAP3 reduced expression group was higher than in the control group, coincident with elevated VEGFA and p-AKT (s473) (Fig. 4e). WB assessment of xenograft tumor tissues revealed that levels of p-AKT (s473), HIF-1α and VEGFA in tumor tissue from the CDK5RAP3 overexpression group was less than in the control group (Fig. 4f), while levels of p-AKT (s473), HIF-1α and VEGFA in tumor tissue from the knockdown group was more than that in the control group (Fig. 4g). Consistent with our in vitro results, these data confirm that decreased expression of CDK5RAP3 promotes tumor growth and angiogenesis in vivo.

### Correlation and prognostic value of CDK5RAP3 and VEGFA in GNEC patients

**Fig. 5:**
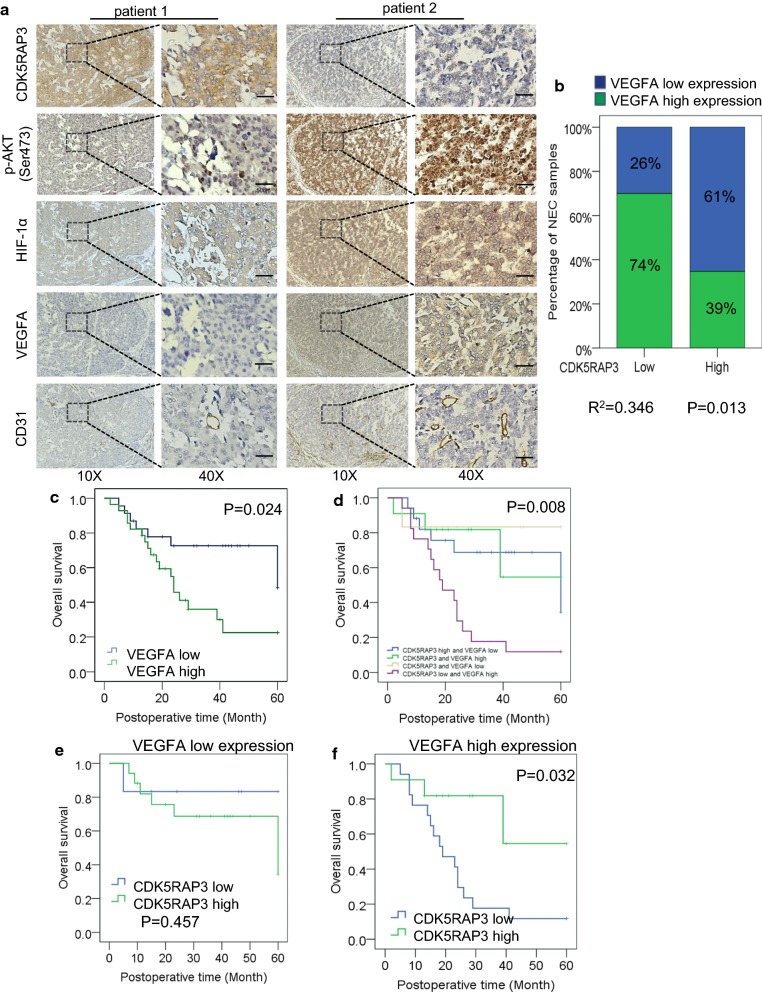
Correlation and prognostic value of CDK5RAP3 and VEGFA in GNEC patients. **a** Representative IHC staining of CDK5RAP3, HIF-1α, p-AKT (S473), CD31 and VEGFA in two GNEC cases (magnification, 10X and 40X, Scale bar, 50 µm). **b** IHC scores of CDK5RAP3 and VEGFA in GNEC tissues. CDK5RAP3 and VEGFA expression are inversely correlated in GNEC tissues (R^2^ = 0.346, P < 0.05, log-rank test). **c** Kaplan–Meier survival curve of gastric cancer patients with “VEGFA high” and “VEGFA low” (P < 0.05, log-rank test) tumors. **d** Kaplan–Meier survival curve of patients with low and high VEGFA expression as well as those with low or high CDK5RAP3 expression (P < 0.05, log-rank test). **e** Kaplan–Meier survival curve of patients with VEGFA low expression and low and high CDK5RAP3 expression (P > 0.05, log-rank test). **f** Kaplan–Meier survival curve of patients with VEGFA high expression and low and high CDK5RAP3 expression (P < 0.05, log-rank test)

To clinically verify the association between CDK5RAP3 and VEGFA, 51 GNEC tissues were subjected to IHC using p-AKT (s473), HIF-1α and VEGFA antibodies. Scoring standards are shown (Additional file 1: Figure S4A). Consistent with our previous results, patients with high expression of CDK5RAP3 exhibited low levels of MVD, p-AKT (s473), HIF-1α and VEGFA, while patients with low expression of CDK5RAP3 exhibited high levels of MVD, p-AKT (s473), HIF-1α and VEGFA (Fig. 5a). IHC scores showed that CDK5RAP3 was negatively correlated with VEGFA expression in patient samples (R^2^ = 0.346, P = 0.013) (Fig. 5b). Overall survival in patients with high VEGFA expression was lower (22.5%) than in patients with low VEGFA expression (48.4%) (Fig. 5c). Next, we determined the prognostic value of combined CDK5RAP3 and VEGFA expression. Patients with low expression of CDK5RAP3 and high expression of VEGFA had the worst prognosis (Fig. 5d). Further stratifying the analysis showed that in patients with low expression of VEGFA, there was no significant difference in overall survival between high and low expression of CDK5RAP3 (Fig. 5e). When VEGFA expression was high, patients with low expression of CDK5RAP3 exhibited poorer prognosis than those with high CDK5RAP3 expression (Fig. 5f). These results indicate that the clinical value of CDK5RAP3 relies on expression of VEGFA. Hence, combined evaluation of CDK5RAP3 and VEGFA is more valuable for patient prognosis.

## Discussion

GNEC is an uncommon malignant gastric cancer. High-density vasculature and distinctly aggressive characteristics convey poor prognosis for this disease. At present, there is a lack of effective treatment options for advanced GNEC [7]. Exploring the molecular basis of invasion and metastasis will provide useful prognostic indicators and effective therapeutic targets for GNEC. In this study, we demonstrated that CDK5RAP3 expression is downregulated in GNEC. Furthermore, low expression of CDK5RAP3 is correlated with more advanced TNM stage, increased tumor microvessel density, and poor prognosis. GNEC cells downregulate CDK5RAP3 expression, resulting in increased VEGFA synthesis and secretion via upregulation of AKT/HIF-1α/VEGFA signaling. We obtained positive and negative results by over-expression and knockdown of CDK5RAP3, compared with the corresponding control groups. Besides, our data revealed that high expression of VEGFA correlates with decreased survival in GNEC patients. CDK5RAP3 expression significantly attenuated the effect of high VEGFA expression on poor prognosis, indicating that the inhibitory effect of CDK5RAP3 on GNEC is closely related to its inhibition of VEGFA expression. Patients with low expression of CDK5RAP3 and high expression of VEGFA showed the worst prognosis. Hence, combined assessment of CDK5RAP3 and VEGFA is significant for predicting patient prognosis in GNEC. Thus, targeting this pathway may represent a promising strategy for the treatment of GNEC.

Extensive studies have shown that CDK5RAP3 is abnormally expressed in a variety of malignancies and participates in the regulation of cell proliferation, apoptosis, transformation and angiogenesis. Wang et al. [29] demonstrated that CDK5RAP3 cooperates with alternative reading frame (ARF) to increase p53 transcriptional activity, thereby leading to G1 cell cycle arrest and inhibition of proliferation. Jiang et al. [30] found that CDK5RAP3 partially inhibits activation of checkpoint kinase 1 and 2 (Chk1 and Chk2) during the DNA damage response and promotes Cdk1 activation and mitotic entry. In addition, Wamsley et al. [31] discovered that CDK5RAP3 directly binds to wild-type p53-induced phosphatase 1 (Wip1) to suppress p38 phosphorylation. Our previous study demonstrated that CDK5RAP3 exerts a tumor suppressive function in gastric cancer by inhibiting β-catenin, a major driver of cancer development [16]. Importantly, we found that CDK5RAP3 controls β-catenin protein expression via activation of AKT to regulate GSK-3β phosphorylation [17]. In HNSCCs, CDK5RAP3 was shown to directly bind to RelA, inhibiting NF-kB transcriptional activity. Decreased CDK5RAP3 expression promotes cellular transformation, xenograft tumor growth, and xenograft tumor vascularity [15]. In this study, we found that CDK5RAP3 expression is also lower in tissues with high MVD compared to tissues with low MVD in GNEC. The same result was further confirmed by xenograft model using ECC12 cells, indicating that low expression of CDK5RAP3 is correlated with increased angiogenesis in GNEC.

Angiogenesis is one of the hallmarks of cancer [32]. When tumor diameter exceeds 2 mm, tissue infiltration cannot maintain its growth, resulting in micro-environmental hypoxia [33] and development of new vessels to provide oxygen and nutrients. In most instances, tumor cells secrete angiogenic substances that initiate the process of angiogenesis [34]. Tumor angiogenesis requires interactions among tumor cells, ECs and mesenchymal cells through growth factors or cytokines and their corresponding receptors [8]. VEGFA is one of the most important growth factors and is related to many signaling pathways, including ERK [35], PI3K/Akt [36], MAPK [37] and endothelial nitric oxide (NO) [38]. These signaling pathways ultimately promote endothelial cell proliferation, migration, invasion, tube formation and vascular permeability, which affect tumor angiogenesis and progression. Previously, we demonstrated that CDK5RAP3 represses AKT phosphorylation, which promotes GSK-3β phosphorylation in gastric cancer [17]. In this study, we further examined the expression of AKT in GNEC cells. Results revealed that CDK5RAP3 also inhibits the phosphorylation of AKT at Ser473, leading to decreased expression of HIF-1α and VEGFA. During the process of angiogenesis, HIF-1α is an important regulatory molecule that induces expression of VEGFA and other angiogenic factors [39, 40]. It has been reported that activation of PI3K/AKT signaling increases VEGFA expression and promotes angiogenesis in multiple tumor types [25–28]. Li et al. [41] found that Akt activation promotes angiogenesis via activation of HIF-1α, which was independent of hypoxia. Therefore, based on our findings and related literature reports, we thought that CDK5RAP3 inhibits angiogenesis in gastric neuroendocrine carcinoma by modulating AKT/HIF-1α/VEGFA signaling. However, more detailed mechanism of how CDK5RAP3 regulates p-AKT, HIF-1α and VEGFA are needed to be explored in the future.

Certain limitations of the present study should be noted. Firstly, the clinical cases used in this study were all retrospective cases. In these patients, we did not retain relevant serological specimens before surgery, so we were unable to provide relevant serum levels. Secondly, there are rare neuroendocrine cancer cell lines in China. ECC12 and ECC10 neuroendocrine cancer cells used in our research were introduced from RIKEN BRC CELL BANK. We don’t have normal neuroendocrine cells in cell banks. We could not compare the expression levels of CDK5RAP3 in these two cell lines with normal. However, we compared the expression of CDK5RAP3 in tumor tissue and adjacent non-tumor tissues by IHC, and found that CDK5RAP3 expression was significantly lower in tumor tissues compared to their respective non-tumor tissues. Finally, general xenograft model maybe not so good for angiogenesis. However, some previous studies had reported that xenograft tumors could be used for detecting the angiogenesis [42, 43]. We also further detected the angiogenesis index of transplanted tumors, and the results showed that there were significant differences in microvessel density in transplanted tumors.

## Conclusions

In conclusion, our study demonstrates that low expression of CDK5RAP3 in GNEC activates the AKT/HIF-1α/VEGFA signaling pathway, increasing secretion of VEGFA from GNEC cells into the tumor microenvironment and promoting tumor angiogenesis. Activation of CDK5RAP3 may represent an alternative approach to inhibiting angiogenesis, thereby providing a novel molecular therapeutic strategy against GNEC.

## Supplementary information

**Additional file 1: Figure S1.** ECC10 and ECC12 identified by Southern blotting and cell immunohistochemistry of SYN and CgA, commonly used diagnostic markers for neuroendocrine carcinoma. (A) Image of ECC10 and ECC12 cells. Southern blotting (B) and immunohistochemistry (C) showing that ECC12 cells are positive for SYN and CgA, ECC10 cells are positive for SYN and negative for CgA, and human gastric cancer cell lines AGS and MGC-803 are negative for both SYN and CgA. Scale bar, 100 µm. (D) The basic expression of CDK5RAP3 from ECC10 and ECC12 was detected by Western blot. (E) The expression of CDK5, p35 and p39 was detected by Western blot. **Figure S2.** CDK5RAP3 in ECC10 cells indirectly inhibits tumor-induced HUVEC migration as shown by wound healing assay. Representative images are shown in (A). Quantification of results is presented in (B). Scale bar, 200 µm. **Figure S3.** CDK5RAP3 decreases VEGFA protein levels in ECC12 cells as shown by immunofluorescence staining (A). Scale bar, 20 µm. **Figure S4.** Immunohistochemical staining of VEGFA expression in GNEC tissues and criteria for immunohistochemistry scoring. Score 0: no staining, Score 1: weak staining, Score 2: moderate staining, Score 3: strong staining. Each section was examined under a high-power field (40X). Scale bar, 50 µm.

## Data Availability

All data generated or analysed during this study are included in this published article.

## Abbreviations

CDK5RAP3/C53: cyclin dependent kinase 5 regulatory subunit associated protein 3
NENs: neuroendocrine neoplasms
GNEC: gastric neuroendocrine carcinoma
VEGFA: vascular endothelial growth factor A
MVD: microvessel density
HUVECs: human umbilical vein endothelial cells
TCM: tumor conditioned medium
HNSCCs: head and neck squamous cell carcinomas
UICC: Union for International Cancer Control
ARF: alternative reading frame
Wip1: wild-type p53-induced phosphatase 1
NO: nitric oxide
IHC: immunohistochemistry
ELISA: enzyme-linked immunoassay
GAPDH: glyceraldehyde-3-phosphate Dehydrogenase
PVDF: polyvinylidene difluoride
BSA: bovine serum albumin
DAB: diaminobenzidine
PBS: phosphate-buffered saline
FBS: fetal bovine serum

## References

[1] Leoncini E, Boffetta P, Shafir M, Aleksovska K, Boccia S, Rindi G (2017). Increased incidence trend of low-grade and high-grade neuroendocrine neoplasms. Endocrine.

[2] Nikou GC, Angelopoulos TP (2012). Current concepts on gastric carcinoid tumors. Gastroenterol Res Pract.

[3] Bosman FT, Carneiro F, Hruban RH (2010). WHO classification of tumours of the digestive system.

[4] Corey B, Chen H (2017). Neuroendocrine tumors of the stomach. Surg Clin N Am.

[5] La Rosa S, Vanoli A (2014). Gastric neuroendocrine neoplasms and related precursor lesions. J Clin Pathol.

[6] Dasari A, Shen C, Halperin D, Zhao B, Zhou S, Xu Y (2017). Trends in the incidence, prevalence, and survival outcomes in patients with neuroendocrine tumors in the United States. JAMA Oncol.

[7] Sorbye H, Welin S, Langer SW, Vestermark LW, Holt N, Osterlund P (2013). Predictive and prognostic factors for treatment and survival in 305 patients with advanced gastrointestinal neuroendocrine carcinoma (WHO G3): the NORDIC NEC study. Ann Oncol.

[8] Lin J, Cao S, Wang Y, Hu Y, Liu H, Li J (2018). Long non-coding RNA UBE2CP3 enhances HCC cell secretion of VEGFA and promotes angiogenesis by activating ERK1/2/HIF-1α/VEGFA signalling in hepatocellular carcinoma. J Exp Clin Cancer Res CR..

[9] Kerbel RS (2008). Tumor angiogenesis. N Engl J Med.

[10] Loizzi V, Del Vecchio V, Gargano G, De Liso M, Kardashi A, Naglieri E (2017). Biological pathways involved in tumor angiogenesis and bevacizumab based anti-angiogenic therapy with special references to ovarian cancer. Int J Mol Sci.

[11] Hoshino Y, Hayashida T, Hirata A, Takahashi H, Chiba N, Ohmura M (2014). Bevacizumab terminates homeobox B9-induced tumor proliferation by silencing microenvironmental communication. Mol Cancer.

[12] Ching YP, Qi Z, Wang JH (2000). Cloning of three novel neuronal Cdk5 activator binding proteins. Gene.

[13] Chen J, Shi Y, Li Z, Yu H, Han Y, Wang X (2011). A functional variant of IC53 correlates with the late onset of colorectal cancer. Mol Med (Cambridge, Mass.).

[14] Stav D, Bar I, Sandbank J (2007). Usefulness of CDK5RAP3, CCNB2, and RAGE genes for the diagnosis of lung adenocarcinoma. Int J Biol Markers..

[15] Wang J, An H, Mayo MW, Baldwin AS, Yarbrough WG (2007). LZAP, a putative tumor suppressor, selectively inhibits NF-kappaB. Cancer Cell.

[16] Wang JB, Wang ZW, Li Y, Huang CQ, Zheng CH, Li P (2017). CDK5RAP3 acts as a tumor suppressor in gastric cancer through inhibition of β-catenin signaling. Cancer Lett.

[17] Zheng CH, Wang JB, Lin MQ, Zhang PY, Liu LC, Lin JX (2018). CDK5RAP3 suppresses Wnt/β-catenin signaling by inhibiting AKT phosphorylation in gastric cancer. J Exp Clin Cancer Res CR..

[18] Wittekind C (2010). TNM system: on the 7th edition of TNM classification of malignant tumors. Pathologe..

[19] Liu L, Bi N, Wu L, Ding X, Men Y, Zhou W (2017). MicroRNA-29c functions as a tumor suppressor by targeting VEGFA in lung adenocarcinoma. Mol Cancer.

[20] Sun C, Li J, Wang B, Shangguan J, Figini M, Shang N (2018). Tumor angiogenesis and bone metastasis—correlation in invasive breast carcinoma. J Immunol Methods.

[21] Fujiwara T, Motoyama T, Ishihara N, Watanabe H, Kumanishi T, Kato K (1993). Characterization of four new cell lines derived from small-cell gastrointestinal carcinoma. Int J Cancer.

[22] Shiimura Y, Ohgusu H, Sato T, Kojima M (2015). Regulation of the human ghrelin promoter activity by transcription factors, NF-κB and Nkx2.2. Int J Endocrinol.

[23] Yoshino N, Ishihara S, Rumi MA, Ortega-Cava CF, Yuki T, Kakazawa S (2005). Interleukin-8 regulates expression of Reg protein in Helicobacter pylori-infected gastric mucosa. Am J Gastroenterol.

[24] Hassan S, Kinoshita Y, Kawanami C, Kishi K, Matsushima Y, Ohashi A (1998). Expression of protooncogene c-kit and its ligand stem cell factor (SCF) in gastric carcinoma cell lines. Dig Dis Sci.

[25] Kang Z, Jiang W, Luan H, Zhao F, Zhang S (2013). Cornin induces angiogenesis through PI3 K-Akt-eNOS-VEGF signaling pathway. Food Chem Toxicol.

[26] Shafee N, Kaluz S, Ru N, Stanbridge EJ (2009). PI3K/Akt activity has variable cell-specific effects on expression of HIF target genes, CA9 and VEGF, in human cancer cell lines. Cancer Lett.

[27] Kazi AA, Molitoris KH, Koos RD (2009). Estrogen rapidly activates the PI3 K/AKT pathway and hypoxia-inducible factor 1 and induces vascular endothelial growth factor A expression in luminal epithelial cells of the rat uterus. Biol Reprod.

[28] Di J, Gao K, Qu D, Yang J, Zheng J (2017). Rap2B promotes angiogenesis via PI3K/AKT/VEGF signaling pathway in human renal cell carcinoma. Tumour Biol.

[29] Wang J, He X, Luo Y, Yarbrough WG (2006). A novel ARF-binding protein (LZAP) alters ARF regulation of HDM2. Biochem J.

[30] Jiang H, Wu J, He C, Yang W, Li H (2009). Tumor suppressor protein C53 antagonizes checkpoint kinases to promote cyclin-dependent kinase 1 activation. Cell Res.

[31] Wamsley JJ, Issaeva N, An H, Lu X, Donehower LA, Yarbrough WG (2017). LZAP is a novel Wip1 binding partner and positive regulator of its phosphatase activity in vitro. Cell Cycle (Georgetown, Tex.).

[32] Hanahan D, Weinberg RA (2011). Hallmarks of cancer: the next generation. Cell.

[33] Viallard C, Larrivée B (2017). Tumor angiogenesis and vascular normalization: alternative therapeutic targets. Angiogenesis.

[34] Khorshidi A, Dhaliwal P, Yang BB (2016). Noncoding RNAs in tumor angiogenesis. Adv Exp Med Biol.

[35] Shin M, Beane TJ, Quillien A, Male I, Zhu LJ, Lawson ND (2016). Vegfa signals through ERK to promote angiogenesis, but not artery differentiation. Development (Cambridge, England)..

[36] Chen HX, Xu XX, Tan BZ, Zhang Z, Zhou XD (2017). MicroRNA-29b Inhibits Angiogenesis by Targeting VEGFA through the MAPK/ERK and PI3 K/Akt Signaling Pathways in Endometrial Carcinoma. Cell Physiol Biochem.

[37] Kim D, Ko HS, Park GB, Hur DY, Kim YS, Yang JW (2017). Vandetanib and ADAM inhibitors synergistically attenuate the pathological migration of EBV-infected retinal pigment epithelial cells by regulating the VEGF-mediated MAPK pathway. Exp Ther Med.

[38] Duval M, Le Boeuf F, Huot J, Gratton JP (2007). Src-mediated phosphorylation of Hsp90 in response to vascular endothelial growth factor (VEGF) is required for VEGF receptor-2 signaling to endothelial NO synthase. Mol Biol Cell.

[39] Wang J, Man GCW, Chan TH, Kwong J, Wang CC (2018). A prodrug of green tea polyphenol (-)-epigallocatechin-3-gallate (Pro-EGCG) serves as a novel angiogenesis inhibitor in endometrial cancer. Cancer Lett.

[40] Park ST, Kim BR, Park SH, Lee JH, Lee EJ, Lee SH (2014). Suppression of VEGF expression through interruption of the HIF-1α and Akt signaling cascade modulates the anti-angiogenic activity of DAPK in ovarian carcinoma cells. Oncol Rep.

[41] Li YM, Zhou BP, Deng J, Pan Y, Hay N, Hung MC (2005). A hypoxia-independent hypoxia-inducible factor-1 activation pathway induced by phosphatidylinositol-3 kinase/Akt in HER2 overexpressing cells. Can Res.

[42] Nowak-Sliwinska P, Alitalo K, Allen E, Anisimov A, Aplin AC, Auerbach R (2018). Consensus guidelines for the use and interpretation of angiogenesis assays. Angiogenesis..

[43] Vermeulen PB, Gasparini G, Fox SB, Colpaert C, Marson LP, Gion M (2002). Second international consensus on the methodology and criteria of evaluation of angiogenesis quantification in solid human tumours. Eur J Cancer..

