# Supplementary figures and images for: CDK5RAP3 inhibits angiogenesis in gastric neuroendocrine carcinoma by modulating AKT/HIF-1α/VEGFA signaling
Source: Cancer Cell Int. 2019 Nov 7;19:282. doi: 10.1186/s12935-019-0997-5 (PMC6839262; doi:10.1186/s12935-019-0997-5)

Figure S1

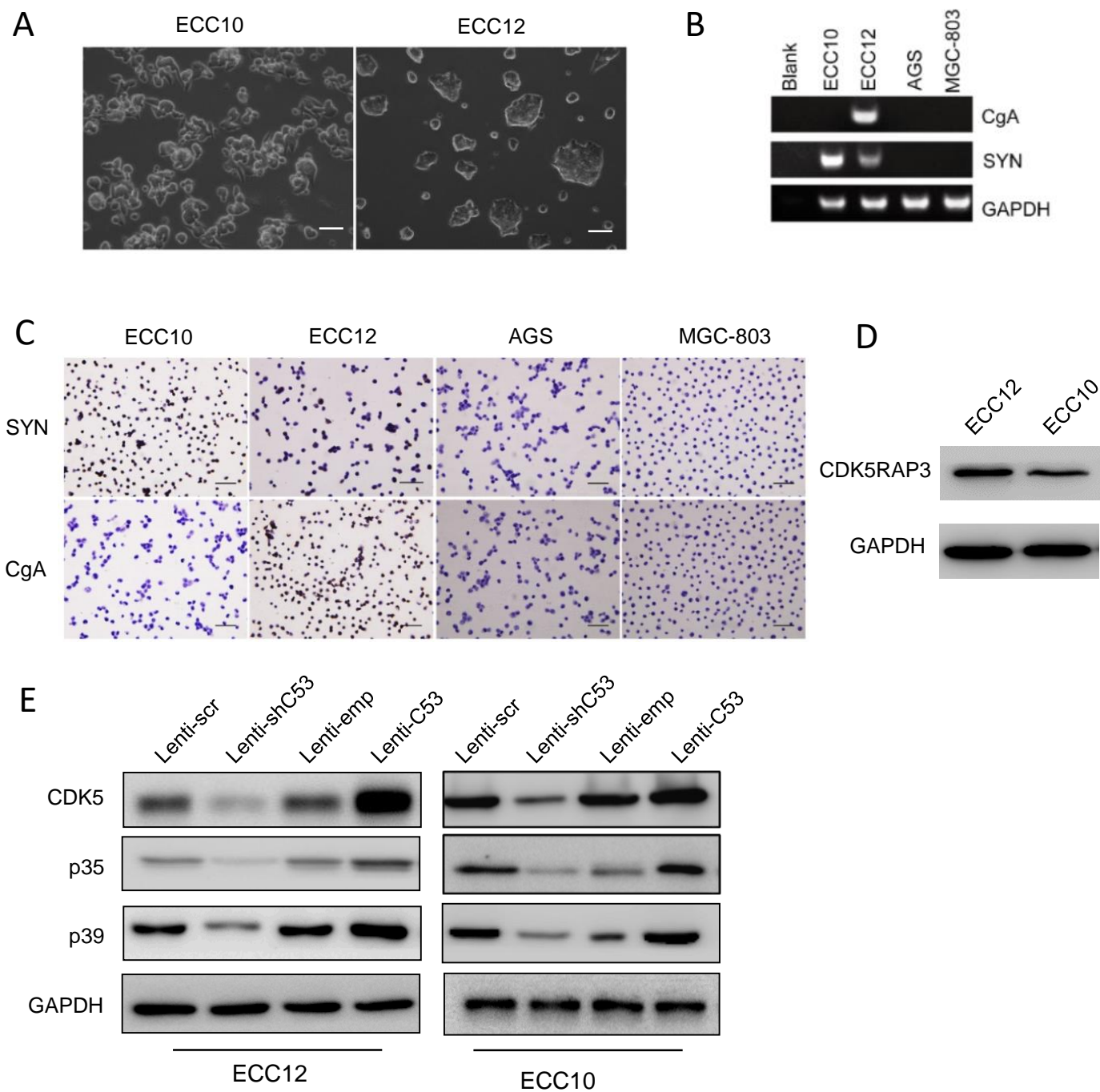

Figure S2

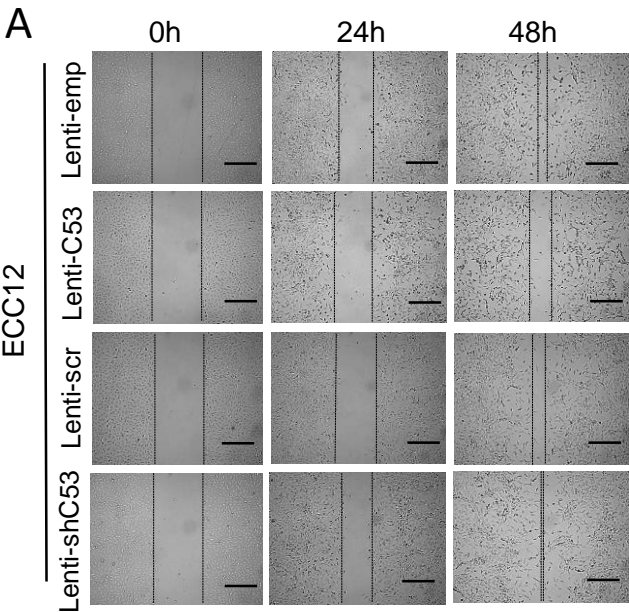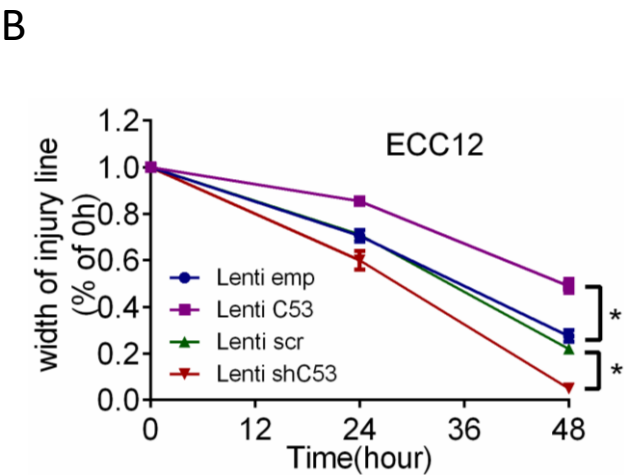

Figure S3

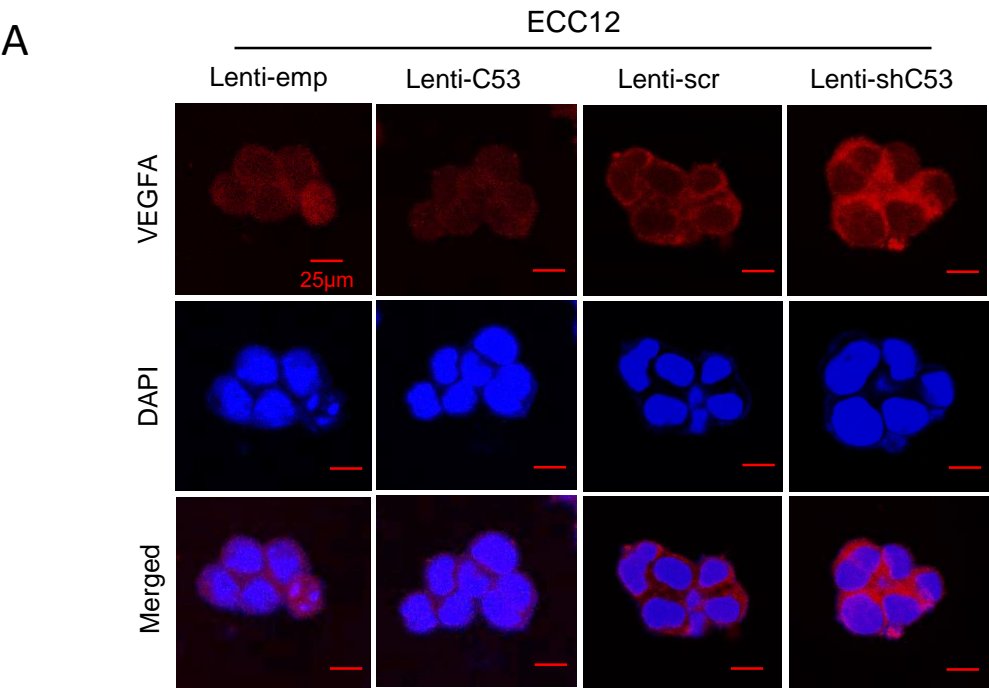

Figure S4

A

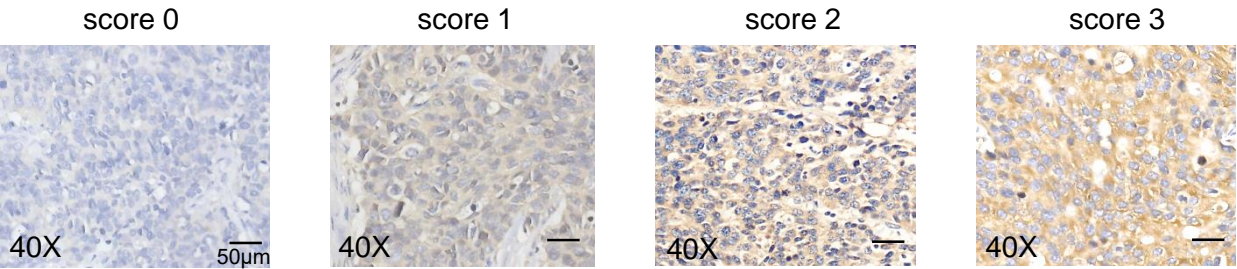

Supplement: Supplementary file 1 — Additional file 1: Figure S1. ECC10 and ECC12 identified by Southern blotting and cell immunohistochemistry of SYN and CgA, commonly used diagnostic markers for neuroendocrine carcinoma. (A) Image of ECC10 and ECC12 cells. Southern blotting (B) and immunohistochemistry (C) showing that ECC12 cells are positive for SYN and CgA, ECC10 cells are positive for SYN and negative for CgA, and human gastric cancer cell lines AGS and MGC-803 are negative for both SYN and CgA. Scale bar, 100 µm. (D) The basic expression of CDK5RAP3 from ECC10 and ECC12 was detected by Western blot. (E) The expression of CDK5, p35 and p39 was detected by Western blot. Figure S2. CDK5RAP3 in ECC10 cells indirectly inhibits tumor-induced HUVEC migration as shown by wound healing assay. Representative images are shown in (A). Quantification of results is presented in (B). Scale bar, 200 µm. Figure S3. CDK5RAP3 decreases VEGFA protein levels in ECC12 cells as shown by immunofluorescence staining (A). Scale bar, 20 µm. Figure S4. Immunohistochemical staining of VEGFA expression in GNEC tissues and criteria for immunohistochemistry scoring. Score 0: no staining, Score 1: weak staining, Score 2: moderate staining, Score 3: strong staining. Each section was examined under a high-power field (40X). Scale bar, 50 µm. [file 12935_2019_997_MOESM1_ESM.pdf]
